# Developmental toxicity and estrogenic activity of antimicrobial phenolic-branched fatty acids using *in silico* simulations and *in vivo* and *in vitro* bioassay

**DOI:** 10.3389/ftox.2024.1380485

**Published:** 2024-09-02

**Authors:** Xinwen Zhang, Helen Ngo, Karen Wagner, Xuetong Fan, Changqing Wu

**Affiliations:** ^1^ Department of Animal and Food Sciences, University of Delaware, Newark, DE, United States; ^2^ U.S. Department of Agriculture, Agricultural Research Service, Eastern Regional Research Center, Wyndmoor, PA, United States

**Keywords:** phenolics, fatty acids, developmental toxicity, chicken embryo, estrogenic activity

## Abstract

Due to the growing safety and environmental concerns associated with biocides, phenolic-soy branched chain fatty acids (phenolic-soy BCFAs) are synthesized as new bio-based antimicrobial agents. Safety evaluation is essential before the wide adoption of these new antimicrobial products. This study was initiated to evaluate the safety of four phenolic-soy BCFAs (with phenol, thymol, carvacrol, or creosote branches). Methyl-branched iso-oleic acid, phenol, and creosote were included in the study as controls. *In silico* toxicity simulation tools predicted that the phenolic BCFAs had much higher toxicities to aquatic organisms than free phenolics did, while the opposite was predicted for rats. The developmental toxicity of four phenolic-soy BCFAs was assessed using an *in vivo* chicken embryonic assay. Results showed that creosote-soy BCFA had much lower mortality rates than creosote at the same dosages. Additionally, creosote-soy BCFA and methyl-branched iso-oleic acid induced minimal estrogenic activity in the concentration range of 10 nM - 1 µM. Carvacrol-soy BCFA treatments significantly increased (*p* < 0.05) oxidative stress levels with higher thiobarbituric acid reactive substances in the livers of chicken embryos. Altogether, the phenolic-soy BCFAs, especially creosote-soy BCFA, reported in this study are potentially promising and safer bio-based antimicrobial products.

## Graphical Abstract

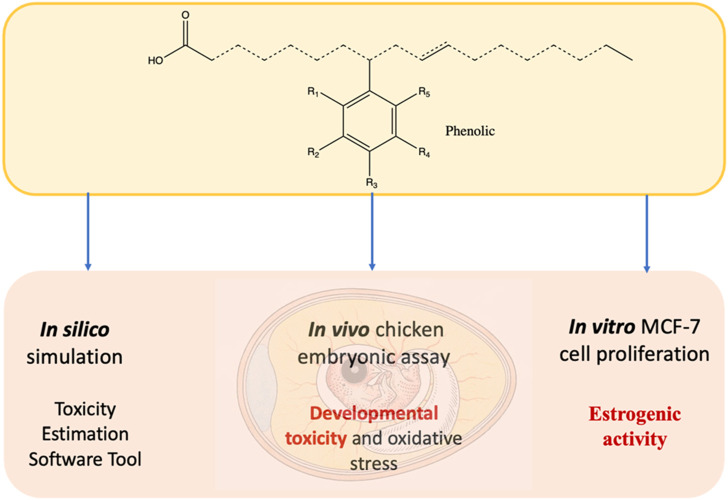

## 1 Introduction

Conventional antimicrobial agents, such as quaternary ammonium compounds, have been widely applied in industrial disinfection, agriculture, and the food industry (Meireles et al., 2017). Nevertheless, due to the growing safety and environmental concerns associated with these biocides, there is a rising need to explore and develop safer natural and bio-based compounds as alternative antimicrobial agents (Gyawali and Ibrahim, 2014). However, even natural and bio-based antimicrobials can pose risks to ecosystems and human health, considering that some natural compounds are reported to have safety concerns. For example, natural phenolics from plant extracts showed genotoxicity in the Comet test (Demma et al., 2009). These compounds could also exhibit estrogenic activity (EA) by interfering with the estrogen signaling pathway, as most phenolics share a similar structure motif with 17β-Estradiol (E2) (Resende et al., 2013). Resende et al. reported the EA of flavonoids, and our group also discovered that carvacrol exerted weak EA at 10^–12^ M (Zhang et al., 2021). Developmental toxicity is another important toxicity endpoint. Flavonoids elicited different levels of adverse effects on the development of zebrafish and chicken embryos (Bugel et al., 2016; Zhang and Wu, 2022). Similar to the phenolic compounds, previous reports have raised concerns about toxicity of lipids, including pro-oxidant activity, mitochondrial toxicity, and aquatic toxicity (Galati and O’brien, 2004; Singh and Chandra, 2019).

Despite all these safety concerns, natural plant monoterpene phenols have been proven to possess antibacterial, antifungal, antioxidant, and anti-inflammatory properties (Khan et al., 2015). For example, the monoterpene phenols carvacrol and thymol are major components of many essential oils and have excellent antimicrobial activity and medicinal properties (Memar et al., 2017). Beechwood creosote consists mainly of natural phenols such as guaiacol and creosol and has been used as preservatives or antiseptics in the pharmaceutical industry (Agency for Toxic Substances and Disease Registry ATSDR, 2024). Natural lipids have also demonstrated antibacterial, antifungal, and anti-inflammatory activities that depend on the chain length and degree of saturation of the fatty acids (FA) (Pohl et al., 2011). For instance, the short-chain lauric acid lipid had excellent antimicrobial activity against viruses and bacteria, while long-chain oleic acid lipid was reported to prevent inflammatory-related and cardiovascular diseases (Matsue et al., 2019; Singh et al., 2020). Some natural antimicrobials are created with balanced benefits and drawbacks, such as ethyl lauroyl arginate (LAE), which is derived from these natural components (lauric acid, L-arginine, and ethanol) and was approved as Generally Recognized as Safe (GRAS) for use as a food preservative (Nübling et al., 2017; Becerril et al., 2013).

**FIGURE 1:**
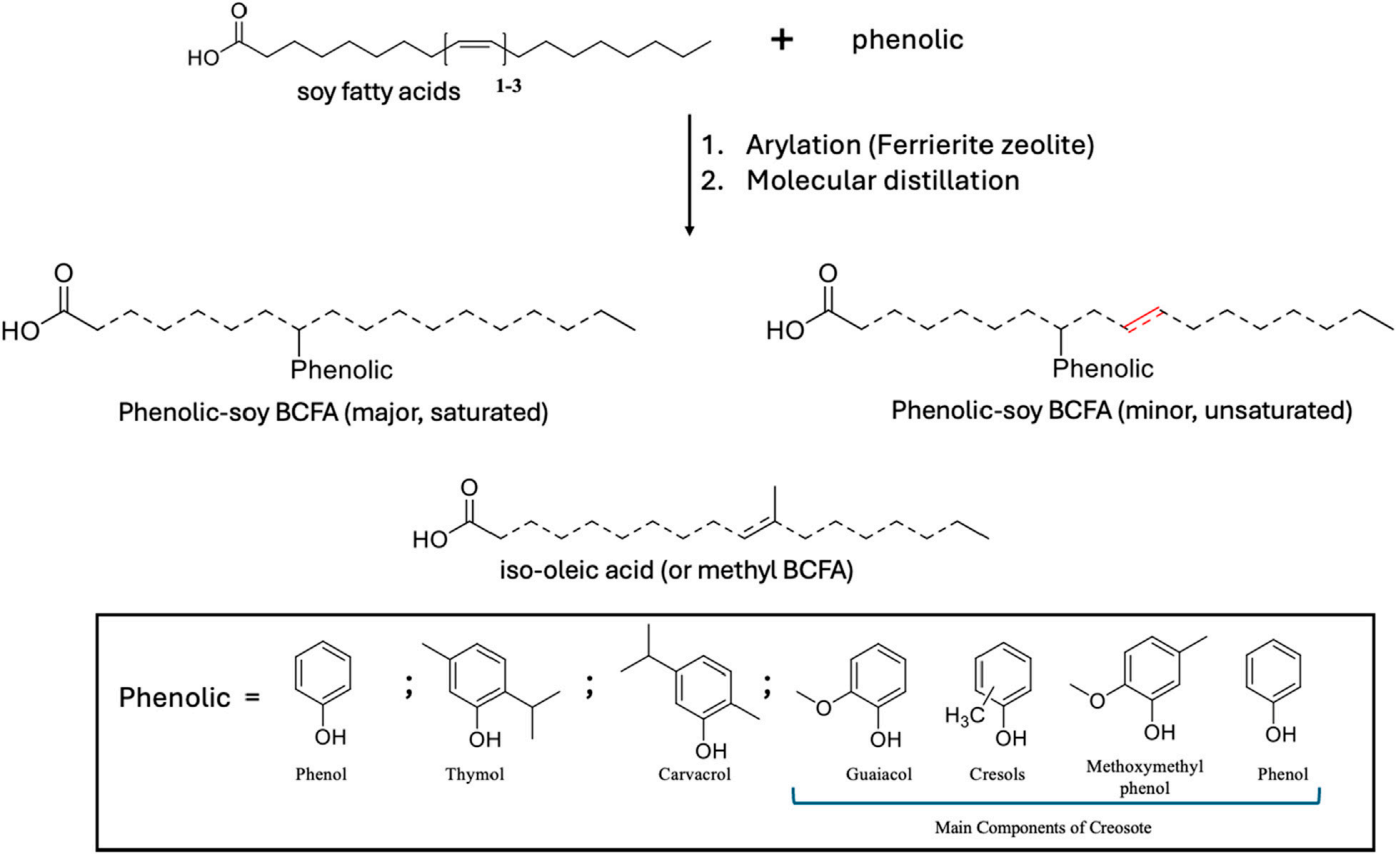
Synthesis of phenolic-soy branched chain fatty acids.

To develop efficient and sustainable antimicrobial agents, our group has recently synthesized a family of bio-based phenolic-branched chain fatty acids (phenolic-BCFAs) products derived from two renewable resources (natural phenolics from plants and fatty acids from vegetable oils). Unlike natural phenolic compounds (*e.g.*, carvacrol, thymol), phenolic-BCFAs do not have strong odors because they are covalently ‘carbon-carbon’ bonded between carbon on the phenolic ring and the carbon double bond of the FA (Ngo et al., 2017; Ngo et al., 2014). Thus, they are highly stable and non-volatile, which is in stark contrast to their parent counterparts. They are also a mixture of different isomers where phenolic groups are attached to various positions of the FA chain (represented as dashed lines in Figure 1). Because of this isomeric characteristic, these products are liquid at room temperature, which is ideal for applications requiring this property (Ngo et al., 2017; Ngo et al., 2014). Additionally, the final phenolic BCFA is a saturated FA product because they were prepared through the arylation process. Although some double bonds (unsaturation) can still be present on the FA chain if the process uses soy FA, the amount of unsaturation is still far less than their parent soy FA (Figure 1). In our early study, these phenolic-BCFAs had excellent properties for inactivating Gram-positive bacteria *(*e.g., *Listeria innocua*), exhibiting much stronger potency than their individual feedstocks (Ngo et al., 2019). In our more recent study, phenol- and creosote- BCFAs showed great promise in synthesizing antibacterial bio-epoxy polymers. The synthesized bio-epoxy polymers, phenol-soy BCFA/ethylene diamine and creosote-soy BCFA/ethylene diamine, showed good antibacterial activity against both Gram-positive and Gram-negative bacteria (Huang et al., 2021).

For these phenolic-soy BCFA products to be widely adapted, there is a critical need to examine their toxicologic profiles. As our earlier research showed that these phenolic-BCFAs were much more potent antibacterial agents than their parent phenolics (Ngo et al., 2019), it is thus important to determine how the toxicological properties of phenolic-BCFA may differ from the properties of the compounds from which they were derived. In this study, several *in silico* simulation tools were conducted as the first step for toxicity prediction because of their cost-effectiveness and screening efficiency compared to experimental studies. Although our phenolic-soy BCFAs are mixtures of different isomers (saturated and unsaturated FA chains), they primarily consist of single-phenolic-soy BCFA isomeric products in the saturated form (Figure 1; Table 1). These single-phenolic-branched FA structures were subjected to the *in silico* simulations. A chicken embryonic assay was used for evaluation of developmental toxicity, as this assay is a promising alternative to traditional *in vivo* models with increasing applications in pharmacological and toxicological studies (Ghimire et al., 2022; Wu et al., 2018). In contrast to rodent animals (*e.g.*, mice and rats), chicken embryos are isolated biological systems of the early life stage of animals. Unlike rodents, chicken embryos are abundant, cost-effective, and easily manipulated (Ghimire et al., 2022). We investigated the developmental toxicity during chicken embryogenesis for phenol, creosote, iso-oleic acid, and four phenolic-soy BCFAs. Iso-oleic acid is a mixture of methyl-branched-chain C_18_-fatty acid isomers and is a well-established lubricant (Ngo et al., 2013) (Figure 1). Furthermore, the potential EA of creosote-soy BCFA was assessed by an MCF-7 cell proliferation assay. To our knowledge, this is the first time that the acute toxicity and developmental toxicity of phenolic-BCFAs are investigated by *in silico, in vitro,* and *in vivo* methods.

**TABLE 1:** Product compositions evaluated by gas chromatography.

Entry	Compound^a^	Phenolic-soy BCFA	Byproduct/
		(wt%)	iso-Oleic acid (wt%)
1	phenol-soy BCFA	94.8	5.2
2	thymol-soy BCFA	90.5	9.5
3	carvacrol-soy BCFA	82.5	17.5
4	creosote-soy BCFA	97.8	2.2

^a^ All compounds are viscous brownish liquid.

## 2 Materials and methods

### 2.1 Materials

Oleic acid (91wt%), thymol (>98.5%), carvacrol (99%), phenol (>99%), and creosote from beechwood tar were purchased from Sigma Aldrich (St. Louis, MO). Ferrierite zeolite (CP914C) from Zeolyst International (Conshohocken, PA US) was calcined for 5 h at 500°C prior to use. Soy oil from a local supermarket was hydrolyzed to generate soy fatty acids. MCF-7 cells were purchased from American Type Culture Collection (ATCC No. HTB-22). Dulbecco’s Modified Eagle Medium (DMEM), phenol red-free DMEM, fetal bovine serum (FBS), charcoal stripped FBS, penicillin-streptomycin (Gibco, 15-140–148), dimethyl sulfoxide (DMSO) (D1391) and phosphate buffered saline (PBS) were purchased from Fisher Scientific (Pittsburgh, PA, US), thiobarbituric acid reactive substance (TBARS) assay kits (Item No. 700870) were purchased from Cayman Chemical Company (Ann Arbor, MI, US).

### 2.2 Preparation of phenolic-soy branched chain fatty acids

Phenolic soy-BCFAs were synthesized and purified as previously reported (Yan et al., 2018). As shown in Figure 1, soy fatty acids, phenolic (individually used), calcined Ferrierite zeolite catalyst, and deionized water were mixed in a high‐pressure mechanical Parr reactor and heated to 260°C for 48 h. After removal of the zeolite catalyst by vacuum filtration, the crude product mixture was subjected to wiped film molecular distillation to isolate fractions that were 83–98 wt% phenolic-soy BCFAs with the remaining byproducts were iso-oleic acid (Table 1). The compounds were characterized using gas chromatography with flame ionization detection (GC/FID), gas chromatography-mass spectrometry with electron ionization (GC-MS-EI), and high-performance liquid chromatography coupled with electrospray ionization-high resolution mass spectrometry (LC–MS-ESI). These methods are discussed in detail in our previous publication (Yan et al., 2018).

### 2.3 Preparation of iso-oleic acid

Iso-oleic acid was synthesized using the modified method of Ngo, *et al* (Ngo et al., 2013). Briefly, oleic acid was combined with triphenylphosphine, distilled water, and a calcined Ferrierite zeolite catalyst in a mechanically stirred pressure Parr reactor at 260°C for 48 h. After the removal of the zeolite by vacuum filtration, the crude product was recrystallized twice with acetone and passed through a wiped film still. The distillate fraction contained 78.6 wt% iso-oleic acid and 21.4 wt% oleic acid and lactones.

### 2.4 Toxicity estimation software tool

The T.E.S.T. (version 5.1) was downloaded from the U.S. Environmental Protection Agency (EPA) website and used to evaluate the toxicity of phenolic BCFAs and their precursors (Epa, 2020). Six toxicity endpoints were included in this study: 96 h fathead minnow LC_50_, 48 h *Daphnia magna* LC_50_, 48 h *Tetrahymena pyriformis* IGC_50_, oral rat LD_50_, developmental toxicity, and Ames mutagenicity. Results were based on a variety of Quantitative Structure Activity Relationship (QSAR) methodologies (hierarchical, FDA, single model, group contribution, and nearest neighbor method).

### 2.5 ProTox-II

The ProTox-II webserver is an open-access tool that utilizes various machine learning models and databases for *in silico* toxicity prediction (Banerjee et al., 2018). The tested phenolic BCFAs and phenolics were inputted as SMILES strings, and the predicted acute toxicity results were generated as predictive LD_50_ values (mg/kg) (Noga et al., 2024).

### 2.6 Kashinhou Tool for Ecotoxicity

Kashinhou Tool for Ecotoxicity (KATE) 2020 version 5.0 was developed by the Health and Environmental Risk Division, National Institute for Environmental Studies (https://kate.nies.go.jp/) and been widely used for ecotoxicology predictions (Melnikov et al., 2016; Furuhama et al., 2010). The SMILES strings of test chemicals are used as inputs and the predicted LC_50_ and (Fish and Daphnid) were generated by using QSAR models with log P.

### 2.7 Chicken embryonic assay

In total, 124 fertilized Leghorn eggs were obtained from the University of Delaware research farm. The eggs were weighed and divided into fifteen groups: vehicle control (VC), and two dosages of each phenol, creosote and four phenolic-soy BCFAs (shown in Table 1) and isooleic acid. On day 6, the eggs were candled, and a hole was drilled for injection of each chemical at two dosage levels, or VC at 0.2 mL. The hole was sealed with Duco Cement, and eggs were placed back in the egg incubator at 38°C and 60% relative humidity. Each test was repeated in two independent trials. The incubation ended on day 18, and the number of dead embryos was recorded during the incubation period. All embryos were dissected and evaluated for deformation, embryo mass, liver mass, heart mass, ratio of embryo to egg weight (REEW), liver somatic index (LSI), and TBARS levels as described in (Zhang and Wu, 2022). Each test was repeated in two independent trials and in duplicate for each trial.

### 2.8 MCF cell proliferation assay

MCF-7 cells were grown and maintained as previously described (Peng et al., 2018). The EA of creosote, creosote-soy BCFA, and iso-oleic acid was investigated at 10 nM to 1 μM. We evaluated EA for newly synthesized phenolic soy BCFAs with a focus on those who have both good performances in antimicrobial activity and low developmental toxicity in the chicken embryo model. Chemicals were dissolved in DMSO and diluted to the test concentrations (0.1% DMSO). No treatment and VC (0.1% DMSO in cell medium) were included as controls. Fresh medium with test compounds was added on day 2 and refreshed every 2 days. After 6-day exposure, cell numbers were measured by MTT assay using a microplate spectrophotometer (Peng et al., 2018). The test was repeated in two independent trials with triplicates for each trial, and cell proliferation rates were calculated for each compound.

### 2.9 Statistical analysis

All data were reported as the mean ± standard deviation (SD) of at least two independent trials. The MCF-7 cell proliferation rates of each compound at different concentrations were compared using a one-way analysis of variance (ANOVA) followed by Dunnett’s test with the statistical software JMP 16.0. The developmental indexes (REEW, LSI, weights) and TBARS values obtained from chicken embryonic assays were evaluated by a one-way ANOVA followed by Dunnett’s test performed with JMP.

## 3 Results

### 3.1 *In silico* simulation study on aquatic toxicity and developmental toxicity

The acute toxicity (Fathead minnow LC_50_, *D. magna* LC_50_, and *T. pyriformis* IGC_50_, and oral rat LD_50_), developmental toxicity, and mutagenicity of phenolic-BCFAs (phenol, thymol, and carvacrol) were predicted by the T.E.S.T via the consensus method (Epa, 2020). We included three structures of each phenolic BCFA: single-phenolic-branched stearic (shown in Figure 1; major, saturated), single-phenolic-branched unsaturated FA (shown in Figure 1; minor, unsaturated), and single-phenolic branch attached at different positions of the FA chain.

With the three selected phenolic-BCFAs evaluated, the T.E.S.T. data show that their Fathead minnow LC_50_, *D. magna* LC_50_, and *T. pyriformis* IGC_50_ values were much lower than the phenolics without the FA attached. This indicates the phenolic-BCFAs have a higher aquatic toxicity. Interestingly, phenolic-BCFAs had higher oral rat LD_50_ values compared to their corresponding phenolic compounds, suggesting lower mammalian toxicities (Table 2). Additionally, all test compounds were negative in the Ames mutagenicity test but were identified as developmental toxins in humans or animals. Compared with saturated phenolic-BCFAs, introducing an additional double bond in the unsaturated structure slightly increased developmental toxicity and the acute toxicity for Fathead minnow, *D. magna*, and *T. pyriformis*. However, the location of the phenolic ring on fatty acids (isomeric aspect) did not affect the simulated toxicity data for phenolic-BCFAs (data not shown).

**TABLE 2:** The estimated toxicity values of phenolic BCFAs and phenols using the T.E.S.T Version 5.1.

Endpoints	Fathead minnow LC_50_ (96 h) mg/L	*D. magna* LC_50_ (48 h) mg/L	*T. pyriformis* IGC_50_ (48 h) mg/L	Oral rat LD50 mg/kg	Developmental toxicity value	Mutagenicity value
Phenol-18:0 (11) FA^a^	0.24	0.83	5.21E-02	2451.47	0.67 (+)	−0.15 (−)
Phenol-18:1 Δ13(11) UFA^b^	0.22	0.68	4.50E-02	5672.09	0.8 (+)	−0.11 (−)
Thymol-18:0 (11) FA	6.41E-02	0.23	1.30E-02	11136.14	0.71 (+)	−0.01 (−)
Thymol-18:1 Δ13(11) UFA	5.91E-02	0.17	1.13E-02	8777.56	0.85 (+)	0.04 (−)
Carvacrol-18:0 (11) FA	6.45E-02	0.21	1.28E-02	11183.67	0.7 (+)	−0.01 (−)
Carvacrol-18:1 Δ13(11) UFA	5.94E-02	0.16	1.11E-02	8814.81	0.84 (+)	0.04 (−)
Phenol	38.69	7.44	131.65	434.02	0.58 (+)	0.25 (−)
Thymol	11.77	3.46	22.6	645.83	0.93 (+)	0.01 (−)
Carvacrol	11.28	2.87	21.33	999.32	0.77 (+)	0.41 (−)

LC_50_ is the concentration of the test chemical in water in mg/L that causes 50% of Fathead minnow or *D*. *magna* to die; IGC_50_ is the concentration of the test chemical in water in mg/L that results in 50% growth inhibition to *Tetrahymena pyriformis*; LD_50_ is the amount of chemical in mg/kg body weight that causes 50% of rats to die after oral ingestion; +means positive; –means negative; UFA: unsaturated fatty acids.

^a^ numbers in parenthesis indicate the location of the phenolic ring on saturated fatty acid chain.

^b^ Δ13 shows the double bond between the 13th and 14th carbons.

Moreover, the other two *in silico* platforms, ProTox-II and KATE, were used to supplement and confirm the simulation results obtained from T.E.S.T. for predicting acute oral toxicity in rats and aquatic toxicity, respectively. Agreed with the findings from T.E.S.T., ProTox-II indicated that phenolic-BCFAs possess a higher oral rat LD50 compared to their corresponding phenolics, and KATE predicted a lower Fish LC50 for phenolic-BCFAs than for phenolics (Table 3).

**TABLE 3:** Predicted toxicity values of phenolic BCFAs and phenols using KATE and ProTox-II.

Chemical	ProTox-II	KATE	
	Oral rat LD_50_ mg/kg	Fish LC_50_ (96 h) mg/L	Daphnid LC_50_ (48 h) mg/L
Phenol-18:0 (11) FA^a^	1,000	0.025	NA
Phenol-18:1 Δ13(11) UFA^b^	1,000	0.033	NA
Thymol-18:0 (11) FA	1,500	0.0017	NA
Thymol-18:1 Δ13(11) UFA	1,500	0.0023	NA
Carvacrol-18:0 (11) FA	1710	0.0017	NA
Carvacrol-18:1 Δ13(11) UFA	1710	0.0023	NA
Phenol	270	45	15
Thymol	640	NA	4.1
Carvacrol	810	2.5	1.5

Fish LC_50_: the concentration which kills 50% of the fish within 96 h exposure.

Daphnid LC_50_: the concentration which immobilizes 50% of the Daphnia within 48 h exposure.

UFA: unsaturated fatty acids.

^a^ numbers in parenthesis indicate the location of the phenolic ring on saturated fatty acid chain.

^b^ Δ13 shows the double bond between the 13th and 14th carbons.

### 3.2 Chicken embryonic assay and developmental toxicity studies

The developmental toxicity of phenolic-soy BCFAs was evaluated at dosages reported for natural phenolics via a chicken embryo model (Zhang et al., 2021; Zhang and Wu, 2022). In the present study, 0.1% DMSO in PBS was used as VC. Shown in Table 4, the embryo mortality and malformation rates of the VC group were 8.3% and 0.0%, respectively, falling within the acceptable range of our lab historical results (mortality rate <10%) and published data. Creosote treatment had the highest mortality rates, at 75% for low-dose group (4.1 μg/kg) and 50% for high-dose group (41 μg/kg). The creosote-soy BCFA treatment showed much lower mortality than creosote, and it had the lowest mortality rates among all the test phenolic-soy BCFA treatments. Phenol-soy BCFA had the second-highest mortality rates among all compounds, with 50% at 145 μg/kg dosage and 25% at 14.5 μg/kg dosage. Notably, no mortality was detected in two phenol treatments. Thymol- and carvacrol-soy BCFAs showed the same mortality rates of 37.5% for the high dosage (180 μg/kg). At the low dosage (18 μg/kg), thymol-soy BCFA had a 20% mortality rate, while no death was observed for carvacrol-soy BCFA. Iso-oleic acid, as a byproduct in phenolic-soy BCFAs only showed a 25% mortality rate in the high dosage group (97 μg/kg). Moreover, deformed embryos (stunting) were observed in the high dosage of phenol-soy BCFA (145 μg/kg), the high dosage of phenol (31 μg/kg), and two iso-oleic acid groups (9.7 and 97 μg/kg).

**TABLE 4:** Mortality rate and malformation rate of chicken embryo on day 18, after injection of thymol-soy BCFA, carvacrol-soy BCFA, phenol-soy BCFA, creosote-soy BCFA, iso-oleic acid, phenol, and creosote at two dosages. For VC groups 12 eggs were used while eight eggs for each treatment group. The number in parentheses represents the number of dead or malformed chicken embryos.

Treatment	VC	Thymol-soy BCFA		Carvacrol-soy BCFA		Phenol-soy BCFA		Creosote-soy BCFA		Iso-oleic acid		Phenol		Creosote	
Dosage (μg/kg)	0.1% DMSO	18.0	180	18.0	180	14.5	145	16.5	165	9.7	97	3.1	31	4.1	41
Mortality rate (%)	8.3 (1)	25.0 (2)	37.5 (3)	0.0 (0)	37.5 (3)	25.0 (2)	50.0 (4)	25.0 (2)	0.0 (0)	0.0 (0)	25.0 (2)	0.0 (0)	0.0 (0)	75.0 (6)	50.0 (4)
Malformation rate (%)	0.0 (0)	0.0 (0)	0.0 (0)	0.0 (0)	0.0 (0)	0.0 (0)	12.5 (1)	0.0 (0)	0.0 (0)	25.0 (2)	12.5 (1)	0.0 (0)	12.5 (1)	0.0 (0)	0.0 (0)

Several developmental indexes were evaluated for the chicken embryos, including embryo and organ weights, REEW, and LSI (shown in Table 5). REEW reflects the growth performance of the embryos after exposure to the treated compounds. Significantly decreased REEW values and embryo weights (*p* < 0.05) were detected in the high-dose phenol-soy BCFA group (145 μg/kg). In the low-dose of iso-oleic acid group, two out of eight embryos were stunted, resulting in a lower REEW value of 0.34 than that of the VC group (0.42) without a significant difference (*p* > 0.05). The LSI (%) indicates a general health status for chicken embryos after exposure to potential pollutants or toxicants. The VC group showed an LSI value of 2.42%, and LSI varied from 2.14% for thymol-soy BCFA to 2.94% for creosote-soy BCFA at low dosage. Similarly, liver and heart weights varied from 0.41 to 0.65 g and 0.17–0.24 g, respectively. These findings suggest that most phenolic-soy BCFAs had no statistically significant differences in the developmental indexes compared with the VC group (*p* > 0.05).

**TABLE 5:** REEW, LSI (%), and weight of embryo and organs of chicken embryos on day 18 after injection of thymol-soy BCFA, carvacrol-soy BCFA, phenol-soy BCFA, creosote-soy BCFA, iso-oleic acid, phenol, and creosote at two dosages. REEW: ratio of embryo to egg weight, LSI: liver somatic index.

Treatment	Dosage (μg/kg)	REEW	LSI (%)	Weights (g)		
				Embryo	Liver	Heart
VC	0.1% DMSO	0.42 ± 0.009	2.42 ± 0.07	22.85 ± 1.07	0.58 ± 0.03	0.23 ± 0.01
Thymol-soy BCFA	18.0	0.43 ± 0.001	2.14 ± 0.08	23.82 ± 0.33	0.51 ± 0.01	0.21 ± 0.02
	180	0.40 ± 0.025	2.62 ± 0.05	22.07 ± 0.92	0.57 ± 0.05	0.19 ± 0.04
Carvacrol-soy BCFA	18.0	0.40 ± 0.001	2.59 ± 0.38	21.36 ± 0.14	0.55 ± 0.07	0.19 ± 0.02
	180	0.37 + 0.005	2.61 ± 0.04	22.13 ± 0.77	0.58 ± 0.01	0.19 ± 0.01
Phenol-soy BCFA	14.5	0.39 ± 0.049	2.67 ± 0.05	22.01 ± 1.82	0.65 ± 0.02	0.23 ± 0.04
	145	0.30 ± 0.073*	2.47 ± 1.06	17.87 ± 4.73*	0.41 ± 0.22	0.17 ± 0.03
Creosote-soy BCFA	16.5	0.42 ± 0.041	2.94 ± 0.27	22.14 ± 1.41	0.65 ± 0.08	0.24 ± 0.02
	165	0.37 ± 0.050	2.79 ± 0.47	20.23 ± 1.35	0.57 ± 0.13	0.21 ± 0.04
Iso-oleic acid	9.7	0.34 ± 0.059	2.65 ± 0.18	19.81 ± 3.33	0.53 ± 0.13	0.22 ± 0.04
	97	0.40 ± 0.037	2.57 ± 0.56	22.33 ± 1.41	0.57 ± 0.08	0.23 ± 0.02
Phenol	3.1	0.39 ± 0.014	2.20 ± 0.25	23.61 ± 2.74	0.52 ± 0.12	0.21 ± 0.03
	31	0.36 ± 0.029	2.23 ± 0.23	22.04 ± 2.54	0.52 ± 0.04	0.22 ± 0.01
Creosote	4.1	0.37 ± 0.007	2.58 ± 0.31	20.31 ± 1.93	0.52 ± 0.01	0.18 ± 0.02
	41	0.41 ± 0.007	2.44 ± 0.39	22.05 ± 1.44	0.54 ± 0.10	0.21 ± 0.01

All values are expressed as mean ± SD, from two independent trials. Differences were evaluated using ANOVA, followed by the Dunnett’s test and statistical significance was indicated by *p* < 0.05.

* means statistically significant difference compared to the VC, group.

**FIGURE 2:**
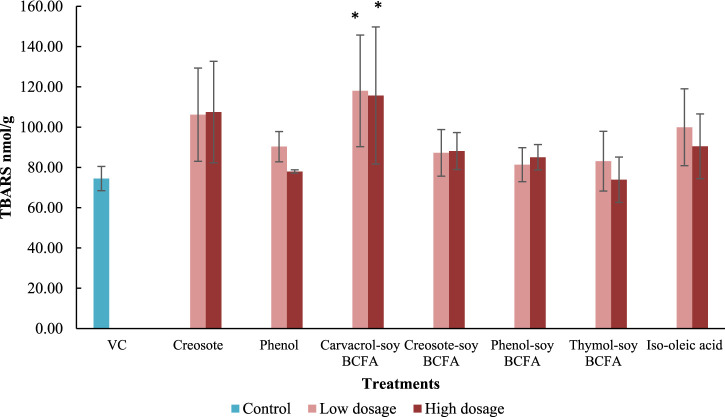
Impacts of creosote, phenol, carvacrol-soy BCFA, creosote-soy BCFA, phenol-soy BCFA, thymol-soy BCFA, and iso-oleic acid on TBARS values. All values are expressed as mean ± SD from two independent trials. Differences were evaluated using one-way ANOVA and followed by the Dunnett’s test, and statistical significance was indicated by *p* < 0.05. * means statistically significant difference compared to the VC group.

Furthermore, the TBARS values were evaluated in fetal livers after treatments. The VC group showed the second-lowest TBARS value at 74.46 nmol/g (Figure 2), just slightly higher than that of the high-dose of thymol-soy BCFA (73.89 nmol/g) group. Significantly increased TBARS values (*p* < 0.05) were only detected at two carvacrol-soy BCFA treatments [115.72 nmol/g (180 μg/kg) and 118.01 nmol/g (18 μg/kg)]. The thymol-soy BCFA, phenol-soy BCFA, and creosote-soy BCFA groups had comparable TBARS values when compared with the findings of VC. Two creosote groups showed similarly TBARS levels (106.16 and 107.48 nmol/g for 4.1 μg/kg and 41 μg/kg dosages, respectively). Additionally, iso-oleic acid at low exposure showed relatively higher (but not significantly) TBARS values at 99.94 nmol/g than VC (*p* > 0.05).

### 3.3 EA effects of phenol and phenol-stearic BCFA, creosote, creosote-soy BCFA, and iso-oleic acid using MCF-7 cell proliferation assay

**FIGURE 3:**
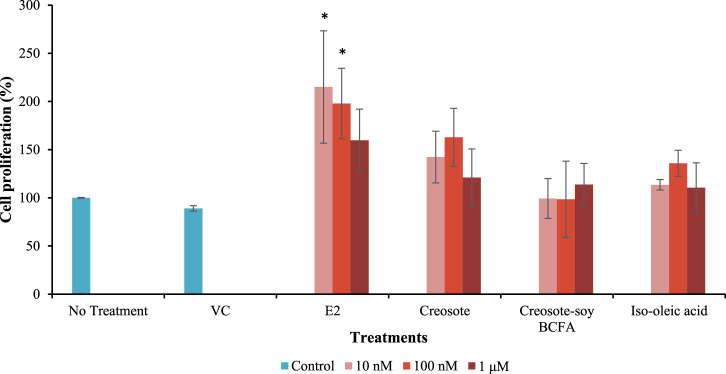
MCF-7 cell proliferation rate after treated with VC, E2, creosote, creosote-soy BCFA, and iso-oleic acid at three concentrations (10 nM–1 μM). Data represented as mean ± SD of two independent trials and significant differences were evaluated using one-way ANOVA and followed by the Dunnett’s test. * means statistically significant difference compared to both no treatment and VC group (*p* < 0.05).

The EA of creosote, creosote-soy BCFA, and iso-oleic acid was further investigated in MCF-7 cells (Figure 3). E2 (positive control) significantly increased cell proliferation rates (*p* < 0.05) at 10 and 100 nM compared with the no treatment and VC groups. Notably, E2 showed a decreased trend in EA as the concentration increased (from 10 nM to 1 μM), which agreed with our previous findings that E2 showed the peak EA at 10^−9^ M (1 nM). Creosote treatments increased the EA level by 162.88% at 100 nM, but without significant differences compared with the VC group (*p* > 0.05). Creosote-soy BCFA and iso-oleic acid exhibited comparable cell proliferation rates at all tested concentrations (*p* > 0.05) compared with VC, showing no EA concerns.

## 4 Discussion

Recently, *in silico* tools based on various QSAR models have been widely used in toxicity prediction as alternatives to traditional *in vivo* methods (RK et al., 2019; Zhou et al., 2020). In this study, we applied three *in silico* tools - T.E.S.T, ProTox-II, and KATE - to predict potential toxicity of three phenolic-soy BCFAs (saturated and unsaturated). Creosote-soy BCFA was not evaluated because the starting material, creosote, is a complex mixture from beechwood tar with at least 36 components, primarily guaiacol, cresols, methoxymethylphenol, and phenol (Figure 1) (Lee et al., 2005). The three tools can only estimate the toxicities of pure compounds; therefore, only the phenol-, thymol-, and carvacrol-soy BCFA were selected. For each of them, three types of pure structures were evaluated: single-phenolic-branched saturated FA (stearic acid), single-phenolic-branched unsaturated FA, and single-phenolic branches attached at different positions of the FA chain.

Our *in silico* findings suggest a discrepancy in toxicity between three aquatic organism models and rats. T.E.S.T. data show that phenolic-BCFAs exhibited much higher aquatic acute toxicity compared to rats, suggesting different toxicity levels and mechanisms between aquatic organisms and rats (mammals) to warrant further study. The extremely lower *T. pyriformis* 50% Inhibition Growth Concentration (IGC_50_) values of three phenolic-BCFAs were related to the inhibitory effect of FAs. FAs have been regarded as a group of pollutants in the aquatic ecosystem and unsaturated fatty acids had more aquatic toxicity concerns (Singh and Chandra, 2019; Wu et al., 2006). Long chain FAs also showed toxicity on microorganisms due to the disruption of the normal function of the cell membrane (Salam et al., 2012). Compared to phenol-BCFA, the thymol- and carvacrol-BCFAs showed higher toxicity on Fathead minnow. Of note, all test compounds were predicted as developmental toxicants from T.E.S.T. which could interfere with normal development of humans or animals. Considering the complex nature of developmental toxicity, further experimental test using a chicken embryonic model was performed in this study.

Chicken embryo is widely used as an alternative animal model within the 3Rs principles for developmental toxicity studies (Ghimire et al., 2022). Since DMSO was used as the solvent for phenolic soy-BCFAs, a limited amount of DMSO (0.1%) without embryotoxicity was administered via egg injection (Luptakova et al., 2021; Hoyberghs et al., 2021). Our previous study showed that carvacrol exhibited higher developmental toxicity in chicken embryos than that of thymol (Zhang et al., 2021). A 45.5% mortality rate was detected for carvacrol at 50 μg/kg dosage, while no death was found at the same treatment concentration for the thymol group. Interesting, carvacrol-soy BCFA in our current study resulted in a lower mortality rate than that of thymol-soy BCFA at the lower dosage (18 μg/kg, Table 4), while at the higher dosage treatment (180 μg/kg) carvacrol-soy BCFA and thymol-soy BCFA showed the same mortality rate. Additionally, our results show that creosote-soy BCFA had the lowest mortality rate among four synthesized phenolic-soy BCFAs, only with a 25% at the lower exposure dosage, although creosote had the most severe deleterious effects at two test dosages (4.1 and 41 μg/kg) on chicken embryo growth. The findings demonstrated that covalently bonding creosote to FA drastically reduced its developmental toxicity. On the other hand, phenol-soy BCFA showed higher adverse effects on chicken embryo growth compared with phenol. Clearly, the choice of phenolic compounds could have significant impacts on the developmental toxicities of the final synthesized phenolic-soy BCFAs and no clear correlation is observed between the toxicities of phenolics and phenolic-soy BCFAs. In other words, developmental toxic phenolics could be used as feedstock to produce phenolic-soy BCFAs with lower or minimal toxicities.

TBARS reflected the lipid peroxidation level in the chicken embryo model and was used as a marker for oxidative damage. The TBARS level in the liver sample varied among different treatments. Carvacrol-soy BCFA at two test dosages significantly increased TBARS values compared with the VC group (*p* < 0.05). Our previous findings also revealed that carvacrol treatment at 50 μg/kg significantly increased TBARS levels in fetal chicken liver, but thymol did not (Zhang et al., 2021). In another study, carvacrol induced reactive oxygen species generation in human fibroblast (WS-1) and gastric adenocarcinoma cells in a dose-dependent manner at 0–100 µM (Günes-Bayir et al., 2018). These findings suggest that the carvacrol branch may play a significant role in the carvacrol-soy BCFA-induced oxidative stress in the liver. Still, there was no clear relationship between the TBARS results and mortality or malformation rates in our study for other three phenolic-soy BCFAs. More insights concerning actual mechanisms remain to be investigated using other methods which might evaluate different pathways other than lipid peroxidation.

Our *in silico* findings were obtained on the purified compounds, while the measured responses from the chicken embryonic assay were recorded from a mixture of two compounds with the composition and purity shown in Table 1, which contributed to some discrepancies between the two methods. Furthermore, when compared with experimental approaches using an animal model, computational tests lacked dose response, generally used simplified models and overlooked the complexity of the life development process. The chicken embryo model can thus shed insights on real toxicity impacts in the early life stage, a vulnerable developmental stage with more toxicity concerns. The purity of three phenolic-soy BCFAs (thymol-soy BCFA, phenol-soy BCFA, creosote-soy BCFA) is more than 90% while that of carvacrol-soy BCFA is over 80% (Table 1). No further purification was performed due to the increased cost. In addition, high antimicrobial activities have already been achieved with these bio-based antimicrobials. Considering their cost-effectiveness and future commercial production, we tested them at current compositions to explore their toxicity and application feasibility.

One of the promising applications of phenolic-soy BCFAs is their use as bio-based antimicrobials in the food industry to control foodborne pathogen contamination with minimal environmental impacts (Ngo et al., 2017; Kazem-Rostami et al., 2023). Although the synthesized phenolic-soy BCFAs demonstrated strong antimicrobial properties against Gram-positive bacteria (*e.g.*, *L. innocua, B. subtilis*, and *E. faecium*) (Fan et al., 2017), their application in the food industry and other fields will be dependent on their potential toxicity. Our results show that the phenolic-soy BCFAs had varying degrees of adverse effects during chicken embryogenesis with different mortality rates. We selected the injection concentrations of phenolic-soy BCFAs at 4.35–54 μg/mL for the chicken embryonic assays. These concentrations were converted to final dosages of 14.5–180 μg/kg (as indicated in Table 4, considering a 0.2 mL injection volume and an average egg weight of 60 g). It is noteworthy that these concentrations were somewhat higher than their minimum inhibitory concentrations (MIC) against *L. innocua* (3.6–7.3 μg/mL) (Ngo et al., 2019), since the embryonic environment might reduce the effectiveness of the phenolic-BCFAs. Using a concentration near the MIC is consistent with the use of *Thymus* extracts or essential oils as a natural antimicrobial preservative in food products at their lowest MIC (Harpaz et al., 2003; Nieto, 2020). Currently, other preservatives such as sodium benzoate may be applied in foods at levels up to 0.1%, or 1,000 μg/mL. Because phenolic-BCFA compounds are effective against Gram-positive bacteria at levels less than 10 μg/mL and have limited aqueous solubility, we anticipate that they would not need to be added at a concentration as high as 1,000 μg/mL.

Furthermore, the potential EA of creosote, creosote-soy BCFA, and iso-oleic acid was investigated via an MCF-7 cell proliferation assay. We chose creosote-soy BCFA as the presentative compound for EA assessment because it demonstrated lower toxicity concerns in the chicken embryonic assay compared with other phenolic BCFAs. The data show that only E2, as the positive control, demonstrated EA at 10 nM and 100 nM. This trend aligns with our previous finding that E2 had the highest EA at 10 nM across the test range from 10^–15^ to 10^–4^ M (Peng et al., 2018). Creosote-soy BCFA, creosote, and iso-oleic acid did not show EA at the three test concentrations. These findings indicate that creosote-soy BCFA (with a good performance in antimicrobial activity) had no EA concern, in addition to its lowest developmental toxicity among all tested phenolic BCFAs.

## 5 Conclusion

This study evaluated the potential developmental toxicities and EA of phenolic soy-BCFAs. Firstly, we predicted the aquatic toxicity, acute toxicity (oral rat LD_50_), developmental toxicity, and mutagenicity of phenolic soy-BCFAs and phenolic compounds using several *in silico* tools (T.E.S.T., ProTox-II, and KATE). The phenolic soy-BCFAs (both saturated and unsaturated) had much higher toxicity in aquatic organisms than free, unreacted phenolics. In rats, the phenolics exhibited higher predicted toxicity than the phenolic soy-BCFAs. All the tested compounds were classified as non-mutagenic developmental toxicants. Acute toxicity and developmental toxicity increased slightly when fatty acids contained a double bond. However, the location of the phenolic branch (structural isomers) had no effect on the *in silico* simulation results. The developmental toxicity of phenolic soy-BCFAs was further investigated by the chicken embryonic assay, which indicated varying levels of developmental toxicity based on the type of phenolic branches. Creosote-soy BCFA had the lowest mortality rate, whereas phenol-soy BCFA showed the highest death rate, malformation number, and altered developmental index (REEW). Thymol and carvacrol soy-BCFAs had comparable mortality rates; however, only carvacrol soy-BCFAs significantly increased the levels of oxidative stress based on TBARS values in the chicken embryonic liver samples. Furthermore, creosote-soy BCFA showed no EA concerns from MCF-7 cell assay. Overall, the phenolic-soy BCFAs, particularly creosote-soy BCFA, presented in this study show promise as potentially safer bio-based antimicrobial products.

## Data Availability

The original contributions presented in the study are included in the article/supplementary material, further inquiries can be directed to the corresponding author.

## Abbreviations

ANOVA, analysis of variance; BCFAs, branched chain fatty acids; DMEM, Dulbecco’s Modified Eagle Medium; DMSO, dimethyl sulfoxide; E2, 17β-Estradiol; EA, estrogenic activity; EPA, Environmental Protection Agency; ERs, estrogen receptors; FAs, Fatty acids; FBS, fetal bovine serum; GRAS, Generally Recognized as Safe; MIC, minimum inhibitory concentrations; LAE, Ethyl Lauroyl Arginate; LSI, liver somatic index; IGC50, 50% Inhibition Growth Concentration; phenolic BCFAs, phenolic-branched chain fatty acids; PBS, phosphate buffered saline; QSAR, quantitative structure-activity relationship; REEW, ratio of embryo to egg weight; SD, standard deviation; TBARS, thiobarbituric acid-reactive substance; T.E.S.T, Toxicity Estimation Software Tool; VC, vehicle control.
